# High Regio‐ and Stereoselective Multi‐enzymatic Synthesis of All Phenylpropanolamine Stereoisomers from β‐Methylstyrene

**DOI:** 10.1002/cbic.202100123

**Published:** 2021-05-13

**Authors:** Maria L. Corrado, Tanja Knaus, Francesco G. Mutti

**Affiliations:** ^1^ Van't Hoff Institute for Molecular Sciences, HIMS-Biocat University of Amsterdam Science Park 904 1098 XH Amsterdam The Netherlands

**Keywords:** alcohol dehydrogenases, Biocatalysis, biocatalytic cascades, chiral amino alcohols, ω-transaminases

## Abstract

We present a one‐pot cascade for the synthesis of phenylpropanolamines (PPAs) in high optical purities (*er* and *dr* up to >99.5 %) and analytical yields (up to 95 %) by using 1‐phenylpropane‐1,2‐diols as key intermediates. This bioamination entails the combination of an alcohol dehydrogenase (ADH), an ω‐transaminase (ωTA) and an alanine dehydrogenase to create a redox‐neutral network, which harnesses the exquisite and complementary regio‐ and stereo‐selectivities of the selected ADHs and ωTAs. The requisite 1‐phenylpropane‐1,2‐diol intermediates were obtained from trans‐ or *cis*‐β‐methylstyrene by combining a styrene monooxygenase with epoxide hydrolases. Furthermore, in selected cases, the envisioned cascade enabled to obtain the structural isomer (1*S*,2*R*)‐1‐amino‐1‐phenylpropan‐2‐ol in high optical purity (*er* and *dr* >99.5 %). This is the first report on an enzymatic method that enables to obtain all of the four possible PPA stereoisomers in great enantio‐ and diastereo‐selectivity.

## Graphical abstract

**Through the simultaneous** combination of stereocomplementary alcohol dehydrogenases and ω‐transaminases, we have developed a cascade for the synthesis of all stereoisomers of nor(pseudo)ephedrines with excellent optical purities and high yields. The cascade requires 1‐phenylpropane‐1,2‐diols as key intermediates, which are obtained from β‐methylstyrenes by combining a styrene monooxygenase with stereocomplementary epoxide hydrolases.

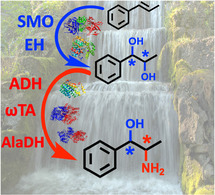

## Introduction

**Scheme 1:**
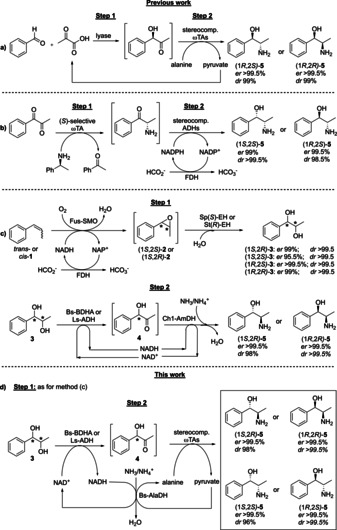
One‐pot enzymatic cascades for the synthesis of optically active phenylpropanolamines. Previous work: a) conversion of benzaldehyde and pyruvate preferably in sequential steps since a concurrent procedure produces ca. 25 % of benzylamine as byproduct;^[4d]^ b) conversion of 1‐phenylpropane‐1,2‐dione in sequential steps comprising de‐activation of ωTA after step 1 to avoid formation of 1‐phenylpropane‐1,2‐diols as by‐products;^[4b]^ c) conversion of *trans‐* or *cis*‐β‐methylstyrene into 1‐phenylpropane‐1,2‐diols followed by biocatalytic hydride‐borrowing amination using an ADH and an AmDH.^[1e]^ This work: d) conversion of 1‐phenylpropane‐1,2‐diols (obtained as in strategy c, step 1) followed by redox‐neutral amination using an ADH, an ωTA and an AlaDH.

Phenylpropanolamines (PPAs) are directly applied as biological active compounds, used as intermediates for the synthesis of APIs, and applied as auxiliaries or ligands in asymmetric organic synthesis.^[1]^ Isolation of PPAs in high optical purity from natural sources is tedious and low yielding,^[2]^ whereas asymmetric chemical and chemo‐enzymatic synthesis still represents a challenge in terms of both selectivities and yields.[^1c^, ^3^] Therefore, a number of fully enzymatic synthesis routes have been developed (see Scheme 1).[^1c^, ^1e^, ^4^] However, a biocatalytic route and related enzymes that enables to obtain all possible PPA stereoisomers (**5**) in high optical purities and yields is currently unavailable. In this context, we have implemented a biocatalytic hydride‐borrowing (HB) cascade for the amination of alcohols into a chemo‐, regio‐ and stereoselective multi‐enzymatic synthesis of PPAs, which involves 1‐phenylpropane‐1,2‐diols (**3**) as the key intermediates (Scheme 1c).^[1e]^ Notably, diols **3** are obtained in high optically pure form from an achiral starting material such as *trans*‐ or *cis*‐β‐methylstyrene (**1**) using a styrene monooxygenase and stereocomplementary epoxide hydrolases.^[1e]^ The subsequent reaction is based on the combination of an alcohol dehydrogenase (ADH) with an amine dehydrogenase (AmDH) in a redox‐neutral transformation.^[5]^ Due to the current scarcity of (*S*)‐selective AmDHs possessing the required substrate scope for PPAs synthesis, only two out of the four isomers of nor(pseudo)ephedrine (**5**) could be attained with this method up to date.^[1e]^ Therefore, in this work, we have investigated a one‐pot enzymatic synthesis of PPAs in which secondary NAD^+^‐dependent ADHs are combined with ω‐transaminases (ωTAs) in another type of redox‐neutral process (Scheme 1d).^[6]^ In this enzymatic network, NAD^+^ coenzyme and alanine are internally recycled by an alanine dehydrogenase from *Bacillus sphaericus* (Bs‐AlaDH) at the expense of ammonia/ammonium species that are provided by the reaction buffer.^[7]^ More in general, ωTAs catalyze the asymmetric transfer of an amino group from an amine donor to a ketone or aldehyde moiety as acceptor through the action of the pyridoxal 5′‐phosphate cofactor (PLP).^[8]^ In this work, six stereocomplementary ωTAs‐namely At(*R*)‐ωTA from *Aspergillus terreus*,^[9]^ As(*R*)‐ωTA from *Arthrobacter* sp.,[^9b^, ^10^] Ac(*S*)‐ωTA from *Arthrobacter citreus*,^[11]^ Cv(*S*)‐ωTA from *Chromobacterium violaceum* (DSM 30191),[^11c^, ^12^] Bm(*S*)‐ωTA from *Bacillus megaterium* SC6394,[^11a^, ^11c^, ^13^] and Vf(*S*)‐ωTA from *Vibrio fluvialis*‐^[14]^ were paired with each of the following NAD^+^‐dependent ADHs such as Aa‐ADH from *Aromatoleum aromaticum*,[^5a^, ^15^] or Bs‐BDHA from *Bacillus subtilis* BGSC1 A1,^[16]^ or Ls‐ADH from *Leifsonia* sp.^[17]^ We investigated the potential of this one‐pot ADH/ωTA cascade for the synthesis of all four stereoisomers of **5**. Interestingly, 1‐amino‐1‐phenylpropan‐2‐ols (**5′**) could also be obtained as PPA structural isomers in selected cases.

## Results and Discussion

### Initial studies

**Scheme 2:**
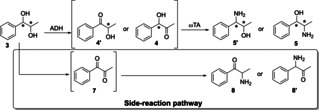
Possible pathways and products for the one‐pot multi‐enzyme cascade.

As described in our previous publication, all of the four stereoisomers of the diol **3** could be obtained in high isolated yields and optical purities via a one‐pot two‐enzyme cascade.^[1e]^ *Cis* and *trans*‐β‐methylstyrene (**1**) were converted into the corresponding epoxides (**2**) by our fused styrene monooxygenase (Fus‐SMO) co‐expressed with a formate dehydrogenase (Cb‐FDH),^[18]^ followed by stereoselective hydrolysis that was catalyzed by stereocomplementary epoxide hydrolases (Sp(*S*)‐EH from *Sphingomonas* sp. HXN200 or St(*R*)‐EH from *Solanum tuberosum*).^[19]^ Thus, (1*S*,2*R*)‐**3** was obtained in 99 % *er* and >99.5 % *dr*; (1*S*,2*S*)‐**3** was obtained in 95.5 % *er* and >99.5 % *dr*; (1*R*,2*S*)‐**3** and (1*R*,2*R*)‐**3** were both obtained in >99.5 % *er* and >99.5 % *dr*. After isolation by extraction, further purification was not required. Herein, starting from these diols **3**, Aa‐ADH from *Aromatoleum aromaticum* was employed to further catalyze the bio‐oxidation of diols (1*S*,2*S*)‐**3**, (1*R*,2*S*)‐**3** and (1*R*,2*R*)‐**3** (SI, Tables S3, S5 and S7). Conversely, Ls‐ADH from *Leifsonia* sp.^[17]^ and Bs‐BDHA from *Bacillus subtilis* BGSC1 A1^[16]^ were employed for the oxidation of diols (1*R*,2*R*)‐**3** and (1*S*,2*R*)‐**3**, respectively (SI, Table S4 and S6). Additionally, we investigated the influence of the temperature on the ADH/ωTA one‐pot cascade as depicted in Scheme 1d using (1*S*,2*S*)‐**3** (5 mM) as test substrate. The reaction was catalyzed by Aa‐ADH combined with either Cv(*S*)‐ωTA (20, 30, 40 and 50 °C) or At(*R*)‐ωTA (30, 40 and 50 °C) in an equimolar ratio (50 : 50 μM). Conversions up to >99 % were observed with all of the tested enzymatic reactions regardless from the applied temperature. However, the best performance in terms of analytical yield of amino alcohol **5** and stereoselectivity was obtained at 30 °C (SI, section 3.2 and Table S2). Depending on the regioselectivity of the applied ADH, two types of structural isomers of 1,2‐amino alcohol can be formed (**5** or **5′**) via intermediates **4** or **4′**, respectively (Scheme 2). Furthermore, another conceivable reaction is the oxidation of both alcohol moieties of substrate **3** to yield diketone **7** that can be subsequently aminated to yield either product **8** or **8′**, depending on the selectivity of the applied ωTA.

### Screening of ωTAs with substrate (1*S*,2*S*)‐3

The first screening of the ωTAs was performed on substrate (1*S*,2*S*)‐**3** (20 mM); (SI section 3.3, Table S3). Aa‐ADH (70 μM) was combined with each of the five stereocomplementary ωTAs (35 μM pure enzyme or 20 mg mL^−1^ lyophilized *E. coli* whole cells in the case of Vf(*S*)‐ωTA). All of the biotransformations were carried out in HCOONH_4_ buffer (pH 8.5, 1 M; 30 °C) supplemented with NAD^+^ (1 mM), PLP (1 mM), d‐ or l‐alanine (5 eq.) and Bs‐AlaDH (20 μM). High total conversions (up to >99 %) were obtained in almost all the cases except when using Vf(*S*)‐ωTA (48 %) and Ac(*S*)‐ωTA (14 %). Moreover, the formation of possible products **5′** and **8′** were not detected with any of the enzymatic systems tested and only traces of **7** (<1 %) were detected. The combination of Aa‐ADH with Cv(*S*)‐ωTA led to 94 % total substrate conversion with the desired vicinal amino alcohol **5** being the main product (86 %). Approximately equal amounts of intermediates **4** and **4′** (4 % and 2 %, respectively) and by‐product **8** (2 %) were detected. We observed similar results for the cascade combining Aa‐ADH and Bm(*S*)‐ωTA. The total conversion of (1*S*,2*S*)‐**3** was 91 % and the vicinal amino alcohol **5** was obtained in 86 % conversion; the intermediates **4** and **4′** and by‐product **8** were again detected in traces (3 %, 2 % and 1 %, respectively). The best performing enzymatic systems were Aa‐ADH/At(*R*)‐ωTA and Aa‐ADH/As(*R*)‐ωTA. Conversions from 98 % up to >99 % of the diol substrate were observed and the main product amino alcohol **5** was obtained in 96 % or 90 % conversion, respectively. The formation of intermediates and by‐products followed the previously reported trend. In contrast, the combination of Aa‐ADH with Vf(*S*)‐ωTA formed the amino alcohol **5** in only 11 %, whereas the main products were intermediate **4** (15 %) and by‐product **8** (13 %). We conclude that Vf(*S*)‐ωTA is not sufficiently active on intermediate **4**, which gets accumulated, partly subsequently oxidized by Aa‐ADH to di‐ketone **7** and then aminated at the latter generated ketone moiety by Vf(*S*)‐ωTA to yield **8**. The use of Ac(*S*)‐ωTA led to the lowest formation of amino alcohol **5** (7 %) with accumulation of intermediate **4**. In summary, the combination of Aa‐ADH with either Cv(*S*)‐, Bm(*S*)‐ At(*R*)‐ or As(*R*)‐ωTA turned out to be fully regioselective (**5** was obtained in all cases while formation of regioisomer **5′** did not occur) and from moderate to high chemoselective (intermediates **4** and **4′** could be detected in tiny amounts). Regarding the stereoselective outcome of the cascade reactions, Cv(*S*)‐ωTA and Bm(*S*‐ωTA formed the product **5** in moderate *er* (86 : 14 [**SS** : RR], for both) and high *dr* (93 : 7 and 95 : 5 [**SS** : RS], respectively). Notably, the highest stereoselectivities were achieved when the two *“R*‐selective” ωTAs (i. e., At(*R*) and As(*R*)) were applied. In fact, the *er* of product **5** was 99 : 1 [**SR** : RS] for both, whereas the *dr* was equal to 98 : 2 and 96 : 4 [**SR** : RR], respectively (Table 1, entries 7 and 8). The slightly imperfect *dr* for these last two enzymatic systems derives from the imperfect *er* (95.5 %) of the applied substrate (1*S*,2*S*)‐**3**‐obtained via stereoselective hydrolysis of the epoxide intermediate^[1e]^ and thus must not be attributed to the inherent selectivity of the alcohol amination cascade.

**Table 1:** Overview of best ADH/ωTA/AlaDH combinations in the one‐pot cascade reaction for the conversion of chiral diols **3** to either optically active **5** or **5′**.

Entry	Substrate	ADH	ωTA	**5** [%]	*er***5**^[a]^ [%]	*dr***5**^[a]^ [%]	**5′** [%]	*er***5′**^[a]^ [%]	*dr***5′**^[a]^ [%]
1	(1*S*,2*R*)‐**3** ^[b]^	Bs‐BDHA	Cv(*S*)	86±3	>99.5 : <0.5 (*SS*)	96 : 4 [*SS* : *RS*]	n.d.	n.a.	n.a.
2	(1*S*,2*R*)**‐3** ^[b]^	Bs‐BDHA	Bm(*S*)	88±1	>99.5 : <0.5 (*SS*)	96 : 4 [*SS* : *RS*]	n.d.	n.a.	n.a.
3	(1*R*,2*R*)‐**3** ^[c]^	Ls‐ADH	Cv(*S*)	76±<1	>99.5 : <0.5 (*RS*)	>99.5 : <0.5 [*RS* : (*RR*/*SS*)]	n.d.	n.a.	n.a.
4	(1*R*,2*R*)‐**3** ^[c]^	Ls‐ADH	Bm(*S*)	81±<1	>99.5 : <0.5 (*RS*)	96 : 2/2 [*RS* : (*SS*/*RR*)]	n.d.	n.a.	n.a.
5	(1*S*,2*R*)‐**3** ^[b]^	Bs‐BDHA	At(*R*)	95±2	>99.5 : <0.5 (*SR*)	98 : 2 [*SR* : *RR*]	n.d.	n.a.	n.a.
6	(1*S*,2*R*)‐**3** ^[b]^	Bs‐BDHA	As(*R*)	92±<1	>99.5 : <0.5 (*SR*)	97 : 3 [*SR* : *RR*]	n.d.	n.a.	n.a.
7	(1*S*,2*S*)‐**3** ^[c]^	Aa‐ADH	At(*R*)	96±<1	99 : 1 (*SR*)	98 : 2 [*SR* : *RR*]	n.d.	n.a.	n.a.
8	(1*S*,2*S*)‐**3** ^[c]^	Aa‐ADH	As(*R*)	90±1	99 : 1 (*SR*)	96 : 4 [*SR* : *RR*]	n.d.	n.a.	n.a.
9	(1*R*,2*R*)‐**3** ^[c]^	Ls‐ADH	At(*R*)	95±<1	>99.5 : <0.5 (*RR*)	>99.5 : <0.5 [*RR* : (*SR*/*RS*)]	n.d.	n.a.	n.a.
10	(1*R*,2*R*)‐**3** ^[c]^	Ls‐ADH	As(*R*)	90±<1	>99.5 : <0.5 (*RR*)	>99.5 : <0.5 [*RR* : (*SR*/*RS*)]	n.d.	n.a.	n.a.
11	(1*R*,2*R*)‐**3** ^[d]^	Aa‐ADH	At(*R*)	21±<1	>99.5 : <0.5 (*RR*)	>99.5 : <0.5 [*RR* : (*SR*/*RS*)]	58±1	>99.5 : <0.5 [*SR* : *RS*]	95 : 5 [*SR* : *RR*]
12	(1*R*,2*R*)‐**3** ^[d]^	Aa‐ADH	As(*R*)	16±<1	>99.5 : <0.5 (*RR*)	>99.5 : <0.5 [*RR* : (*SR*/*RS*)]	61±<1	>99.5 : <0.5 [*SR* : *RS*]	>99.5 : <0.5 [*SR* : *RR*/*SS*]

n.d.=not detected; n.a.=not applicable. [a] Determined by RP‐HPLC analysis, after derivatization with GITC (only observed isomers were reported). [b] 15 mM. [c] 20 mM. [d] 10 mM. The reported values represent the average of two samples.

### Screening of ωTAs with substrate (1*R*,2*R*)‐3

The same type of screening was performed with substrate (1*R*,2*R*)‐**3** (20 mM) (SI section 3.4, Table S4). Ls‐ADH (35 μM) was paired with each of the ωTAs (70 and 50 μM, respectively) and high total conversions ranging from 88 % up to >99 % were achieved, the only exception being the combination with Ac(*S*)‐ωTA that resulted in a total substrate conversion of 33 % and a partial conversion into amino alcohol **5** of 22 %. The combination of Ls‐ADH with Vf(*S*)‐ωTA led to full conversion of the substrate, but without any formation of product **5**. The main products were intermediates **4** (54 %), **4′** (13 %) and by‐product **8** (33 %). In contrast, with all the other enzymatic combinations, compounds **4**, **4′**, **7** and **8** were only observed in traces. Furthermore, as for the conversion of substrate (1*S*,2*S*)‐**3**, only the amino alcohol **5** was formed while neither product **5′** nor by‐product **8′** were detected in any of the tested conditions. The combination of Ls‐ADH with Cv(*S*)‐ωTA for the conversion of substrate (1*R*,2*R*)‐**3** resulted in 88 % total conversion and 76 % formation of amino‐alcohol **5**. Similar results were observed when applying the “*S*‐selective” Bm(*S*)‐ωTA (90 % overall conversion, 81 % of **5**). Again, the best performance was observed with the two “*R*‐selective” ωTAs (*i. e*., At(*R*) and As(*R*)) that exhibited quantitative conversion and the main product was the amino alcohol **5** (95 % and 90 % partial conversion, respectively). Notably, regarding the stereoselective outcome of the cascade reactions, product **5** was obtained in high *er* (>99.5 : <0.5 [**RS** : SR] using Cv(*S*)‐ and Bm(*S*)‐ωTA or >99.5 : <0.5 [**RR** : SS] using At(*R*) and As(*R*)‐ωTA) and *dr* (>99.5 : <0.5 [**RS** : (RR+SS)] using Cv(*S*)‐ωTA or >99.5 : <0.5 [**RR** : (SR+RS)] using At(*R*) and As(*R*)‐ωTA; for selection see Table 1, entries 3, 4, 9 and 10; for full dataset, see Table S4). These higher stereoselectivities than the previously reported ones for the reactions with substrate (1*S*,2*S*)‐**3** catalyzed by Aa‐ADH derive from the superior selectivity of Ls‐ADH toward (1*R*,2*R*)‐**3**.

Based on preliminary results in which the formation of the regioisomer **5′** was observed, (1*R*,2*R*)‐**3** (10 mM) was also tested for the enzymatic one‐pot cascade catalyzed by Aa‐ADH (50 μM) combined with the ωTAs (50 μM) as reported in Table S5 (SI section 3.4). Conversions ranging from 20 to 76 % were observed with all tested “*S*‐selective” ωTAs and leading to the sole formation of product **5** (7–26 %). Accumulation of intermediates **4** and **4′** was also observed and it was highest for the Aa‐ADH/Vf(*S*)‐ωTA combination (61 % of **4′**). Only trace amounts of compound **7** and by‐product **8** were formed. In general, the cascade reactions proceeded with elevated regio‐ and stereo‐selectivities, thus yielding product **5** in high *er* (>99.5 : <0.5 [**RS** : SR]) and *dr* (>99.5 : <0.5 [**RS** : (SS+RR)]) with the only exception being the combination with Bm(*S*)‐ωTA (*dr* 86 : 14 [**RS** : RR]). Aa‐ADH/At(*R*)‐ωTA and Aa‐ADH/As(*R*)‐ωTA were the most notable enzymatic combinations that resulted in high conversions (94–97 %) although both regioisomers **5** and **5′** were formed (Table 1, entries 11 and 12). Using At(*R*)‐ωTA, the composition of the products mixture was **5′** (58 %), **5** (21 %) and **8′** (13 %); traces of intermediates **4′** (2 %) and by‐product **8** (1 %) were also detected. Comparable results were obtained by combining Aa‐ADH and As(*R*)‐ωTA. Nevertheless, both enzymatic cascades exhibited elevated *er* and *dr* for both the regioisomer products (**5′**: *er* >99.5 : <0.5 [**SR** : RS] and *dr* up to >99.5 : <0.5 [**SR** : (RR+SS)]; **5**: *er* >99.5 : <0.5 [**RR** : SS] and *dr* >99.5 : <0.5 [**RR** : (SR+RS)]). Based on the known stereoselectivities of the enzymes employed in the cascades, the expected stereochemistry of product **5** and **5′** was verified in all cases. For product (1*S*,2*R*)‐**5′**, one must consider the occurrence of the switch of the Cahn‐Ingold‐Prelog (CIP) priority.

### Screening of ωTAs with substrate (1*S*,2*R*)‐3

The conversion of (1*S*,2*R*)‐**3** (15 mM) was conducted by combining Bs‐BDHA (50 μM) and each of the stereocomplementary ωTAs (50 μM) (SI section 3.5, Table S6). High conversions (92–98 %) were observed with all tested enzymatic cascades except for the reactions comprising either Vf(*S*)‐ωTA (29 %) or Ac(*S*)‐ωTA (16 %). Moreover, only product **5** (i. e., no formation of **5′**) was observed for all reactions. By‐product **8′** was never detected and intermediate **7** was only observed in traces (<1 %) in all the tested reactions. Using Cv(*S*)‐ωTA, the cascade proceeded with 92 % total conversion and product **5** was formed in 86 % conversion, while only traces (1–3 %) of intermediates **4** and **4′**, and by‐product **8** were detected (Table 1, entry 1). Similar results were observed with Bm(*S*)‐ωTA that resulted in a total conversion up to 94 % and formation of product **5** in 88 % conversion (Table 1, entry 2). On the other hand, both Vf(*S*)‐ωTA and Ac(*S*)‐ωTA led to low conversion and the formation of the amino alcohol **5** was mediocre (3 % and 6 %, respectively). In the case of Vf(*S*), both intermediates **4** and **4′** (11 % and 6 %) and the by‐product **8** (8 %) were detected. For the Ac(*S*) cascade, only intermediate **4** was detected in 4 % along with traces of **4′** and **8** (<1 %). Finally, Bs‐BDHA/At(*R*)‐ωTA and Bs‐BDHA/As(*R*)‐ωTA were the best performing combinations that led to nearly quantitative conversions (98 % and 97 %, respectively) with amino alcohol **5** being the main product (95 % and 92 %) and intermediates **4** and **4′** and by‐product **8** being the other components (1–3 %) of the product mixture (Table 1, entries 5 and 6). By using Cv(*S*)‐, Bm(*S*)‐, At(*R*)‐ and As(*R*)‐ωTA, a high regio‐ and chemo‐selectivity was obtained. Moreover, the stereoselective outcome of the reaction was excellent as the amino alcohol **5** was always formed with elevated *er* (>99.5 : <0.5 [**SS** : RR] or >99.5 : <0.5 [**SR** : RS]) and *dr* (96 : 4 [**SS** : RS] or 98 : 2 [**SR** : RR]); the slightly lower *dr* stemmed again from the imperfect *er* of substrate (1*S*,2*R*)‐**3** used, which was previously obtained from *trans*‐**1** using Fus‐SMO and an epoxide hydrolase.

### Screening of ωTAs with substrate (1*R*,2*S*)‐3

Finally, substrate (1*R*,2*S*)‐**3** (10 mM) was converted by Aa‐ADH (70 μM) paired with each of the selected stereocomplementary ωTAs (35 μM) (SI section 3.6, Table S7). High conversions (from 87 % up to >99 %) were achieved for all the tested enzymatic cascades, the exception being the Aa‐ADH/Ac(*S*)‐ωTA combination that yielded 23 % conversion (10 % formation of **5**). In the case of Cv(*S*)‐ωTA, amino alcohol **5** (77 % formation) was the main product and formation of its regioisomer **5′** did not occur. The other components of the reaction mixture were intermediates **4** (1 %), **4′** (3 %), **7** (2 %) and by‐product **8** (10 %). Similar results were detected for the enzymatic system Aa‐ADH/Bm(*S*)‐ωTA. Although the cascade combination of Aa‐ADH with Vf(*S*)‐ωTA exhibited very high conversion (99 %), only 4 % of the amino alcohol product **5** was formed. The major products were intermediate **4** (33 %), its aminated counterpart **8** (45 %) and intermediate **4′** (13 %). Furthermore, traces of **5′** (1 %) and **7** (1 %) were also observed. The cascade reactions comprising one of the two “*R*‐selective” ωTAs, namely At(*R*)‐ωTA or As(*R*)‐ωTA, yielded full substrate conversion and high partial conversion into the amino alcohol **5** (87 % and 84 %, respectively). In the At(*R*)‐ωTA catalyzed reaction, we did not detect intermediates **4**, **4′** and **7** but we observed by‐products **8** and **8′** (4 % each). As(*R*)‐ωTA showed an equal distribution of intermediates **4**, **4′** and **7** (1 %) and by‐product **8** was formed in 13 % conversion. A bit surprisingly, although substrate (1*R*,2*S*)‐**3** possesses 1*R* configuration, only trace amounts of the amino alcohol **5′** were obtained (up to 5 %) when Aa‐ADH was combined with either At(*R*)‐ωTA or As(*R*)‐ωTA. In contrast, the 1*R* configuration has previously exhibited a beneficial behavior in the conversion of substrate (1*R*,2*R*)‐**3** to the related amino alcohol **5′**. In general, in this latter screening, product **5** was still obtained in elevated *er* (>99.5 : <0.5 [**RS** : SR] or >99.5 : <0.5 [**RR** : SS]) albeit with low to moderate *dr* (max 83 %).

## Overview

Table 1 provides an overview of the best combinations of ADHs and ω‐TAs for the one‐pot conversion of enantiopure diols **3** to the targeted amino alcohols **5**. Moreover, the enzymatic cascades that enabled the access to the regioisomer (1*S*,2*R*)‐**5′** are also reported.

**Table 2:** Multi‐enzyme conversion of *trans*‐ and *cis*‐**1** into (1*S*,2*S*)‐**5** and (1*R*,2*S*)‐**5** through two consecutive one‐pot transformations.

Entry	Sub.	Step 1 yield [%]	Step 2 yield [%]	Combined yield [%]	*er***5** [%]	*dr***5** [%]
1	*trans*‐**1**	86	83	71	>99.5 : <0.5 (1*S*,2*S*‐**5**)	97 : 3^[a]^
2	*cis*‐**1**	70	75	53	>99.5 : <0.5 (1*R*,2*S*‐**5**)	>99.5 : <0.5

[a] Dependent on stereoselectivity of Step 1.

### Preparative scale reactions

To enable efficient synthesis of the final products, we started the multi‐enzyme process from inexpensive *trans*‐**1** (296 mg) and *cis*‐**1** (296 mg) that were converted into (1*S*,2*R*)‐**3** and (1*R*,2*R*)‐**3** in 86 % and 70 % isolated yield, respectively (Table 2; for details and procedure, see SI section 4.1 and Table S8). Enantiomerically pure diols were extracted and directly used for the next one‐pot transformation. We performed the hydride‐borrowing biocatalytic amination using either (1*S*,2*R*)‐**3** (0.69 mmol, 106 mg) with Bm(*S*)‐ωTA (50 μM) and Bs‐BDHA (50 μM) or (1*R*,2*R*)‐**3** (1.3 mmol, 202 mg) with Cv(*S*)‐ωTA (70 μM) and Ls‐ADH (35 μM). Bs‐AlaDH (20 μM) was used as l‐alanine and NAD^+^‐recycling enzyme in both cases. Thus, (1*S*,2*S*)‐**5** and (1*R*,2*S*)‐**5** were obtained with similar yields (83 % and 75 %, respectively) and stereoselectivity as for the analytical scale reactions (for details, see SI section 4.2). Table 2 reports the yields for the consecutive one‐pot reactions (step 1: diol formation; step 2: hydride‐borrowing amination), the yields for the combined steps and the optical purity of the obtained PPAs product.

## Conclusion

We have developed a one‐pot enzymatic cascade in which a panel of secondary NAD^+^‐dependent ADHs was combined with a panel of ωTAs to convert chiral 1,2‐diols **3** into all of the four possible stereoisomers of phenylpropanolamine **5**. The requisite chiral **3** were enzymatically synthesized in a one‐pot cascade catalyzed by a Fus‐SMO combined with one of two stereocomplementary EHs. (1*S*,2*S*)‐**5** was obtained in a maximum of 88 % yield and high optical purity (*er* >99.5 %; *dr* 96 %) by combining Bs‐BDHA with Bm(*S*)‐ωTA. (1*R*,2*S*)‐**5** was obtained in 76 % yield and perfect optical purity (*er* >99.5 %; *dr* >99.5 %) by combining Ls‐ADH with Cv(*S*)‐ωTA. (1*S*,2*R*)‐**5** was obtained in 95 % yield and high optical purity (*er* >99.5 %; *dr* 98 %) by combining Bs‐BDHA with At(*R*)‐ωTA. (1*R*,2*R*)‐**5** was obtained in a maximum of 95 % yield and perfect optical purity (*er* >99.5 %; *dr* >99.5 %) by combining Ls‐ADH with At(*R*)‐ωTA. Additionally, as a proof‐of‐principle, we proved that this enzymatic strategy is also suitable to yield the structural isomers **5′**. In particular, we converted (1*R*,2*R*)‐**3** into (1*S*,2*R*)‐**5′** in 58–61 % partial conversions and high optical purity (*er* and *dr* >99.5) by combining Aa‐ADH with either At(*R*)‐ωTA or As(*R*)‐ωTA. In summary, the one‐pot cascade reported in this work is currently the only available enzymatic method that enables to obtain all of the four possible PPAs stereoisomers in great enantio‐ and diastereo‐selectivity. It also provides high yields, thus greatly expanding the repertoire of chemical and enzymatic methods for the synthesis of optically pure phenylpropanolamines.

## Experimental Section

List of enzymes with details, procedures for preparations of enzymes, procedures for cascade reactions and methods for analytical determinations are reported in the Supporting Information.

## Conflict of interest

The authors declare no conflict of interest.

## Supporting information

As a service to our authors and readers, this journal provides supporting information supplied by the authors. Such materials are peer reviewed and may be re‐organized for online delivery, but are not copy‐edited or typeset. Technical support issues arising from supporting information (other than missing files) should be addressed to the authors.

Supplementary
