# Supplementary material for: High Regio‐ and Stereoselective Multi‐enzymatic Synthesis of All Phenylpropanolamine Stereoisomers from β‐Methylstyrene
Source: Chembiochem. 2021 May 13;22(13):2345–50. doi: 10.1002/cbic.202100123 (PMC8359840; doi:10.1002/cbic.202100123)
Supplement: Supplementary file 1 — Supplementary [file CBIC-22-2345-s001.pdf]

ChemBioChem

Supporting Information

High Regio- and Stereoselective Multi-enzymatic Synthesis of All Phenylpropanolamine Stereoisomers from β-Methylstyrene

Maria L. Corrado, Tanja Knaus, and Francesco G. Mutti*

Supporting Information for:

High regio- and stereoselective multi-enzymatic synthesis of all phenylpropanolamine stereoisomers from β-methylstyrene

Maria L. Corrado,[a] Tanja Knaus,[a] and Francesco G. Mutti[a]*

[a]Van 't Hoff Institute for Molecular Sciences, University of Amsterdam, HIMS-Biocat, Science Park 904, 1098 XH Amsterdam, the Netherlands.

* Corresponding author: f.mutti@uva.nl

Table of Contents

1. List of abbreviations ... 2

2. Enzymes used in this study ... 3

2.1 General procedures for expression and purification of enzymes ... 3

3. Optimization of the one-pot cascade for the conversion of chiral diols 3 to optically active amino alcohols 5 and 5' by ADHs coupled with ω-transaminases ... 5

3.1 General procedure for the one-pot cascade for the conversion of enantiopure diols **3** catalyzed by ADHs combined with ωTAs ... 5

3.2 Temperature study for the conversion of (1S,2S)-**3** catalyzed by Aa-ADH combined with Cv(S)-ωTA or At(R)-ωTA ... 5

3.3 Screening of Aa-ADH with a panel of ω-transaminases with substrate (1S,2S)-**3** ... 6

3.4 Screening of Ls-ADH and Aa-ADH with a panel of ω-transaminases with substrate (1R,2R)-**3** ... 7

3.5 Screening of Bs-BDHA with a panel of ω-transaminases with substrate (1S,2R)-**3** ... 8

3.6 Screening of Aa-ADH with a panel of ω-transaminases with substrate (1R,2S)-**3** ... 9

4. Preparative scale reactions ... 10

4.1 One-pot biocatalytic synthesis of enantiomerically pure diols (1S,2R)-3 and (1R,2R)-3 from cis- and trans-β-methylstyrene at hundreds of milligrams scale ... 10

4.2 One-pot biocatalytic amination of (1R,2R)-3 and (1S,2R)-3 at hundreds of milligrams scale ... 10

5. Analytics ... 12

5.1 Methods ... 12

5.2 Representative GC-FID and RP-HPLC spectra ... 13

6. References ... 16

1. List of abbreviations

ADH	Alcohol dehydrogenase
AlaDH	Alanine dehydrogenase
BDHA	2,3-Butanediol dehydrogenase
dr	Diasteromeric ratio
EDTA	Ethylenediaminetetraacetic acid
EH	Epoxide hydrolase
er	Enantiomeric ratio
FDH	Formate dehydrogenase from *Candida boidinii*
Fus-SMO	Styrene monooxygenase fusion enzyme
GITC	2,3,4,6-Tetra-O-acetyl-β-D-glucopyranosyl isothiocyanate
$HCOONH_4$	Ammonium formate buffer
KH_2PO_4	Potassium dihydrogen phosphate
KPi	Potassium phosphate
LB	Luria-Bertani medium
MTBE	*tert*-Butyl methyl ether
n.d.	Not detected
n.m.	Not measured
NaCl	Sodium chloride
$NAD(P)^+$	Nicotinamide adenine dinucleotide (phosphate)
PLP	pyridoxal 5'-phosphate
RP-HPLC	Reverse-phase HPLC
rr	Regioisomeric ratio
SDS-PAGE	Polyacrylamide gel electrophoresis
TA	Transaminase
Tris-HCl	Tris(hydroxymethyl)aminomethane buffer

General Information. Enzymatic synthesis of the diol substrates, the selection of the secondary NAD^+-dependent ADHs and the chemical synthesis of amino alcohol references **5** and **5'** are reported elsewhere.[1] All the enzymes used in this study are reported in Table S1 together with the source and expression conditions.

2. Enzymes used in this study

Table S1. Source and expression conditions for enzymes used in this study. For the definition of enzyme classes, see the list of abbreviations.

Name	Source/Comment	Plasmid	Tag	Expression/Purification	Used form	Ref
Fus-SMO (1) + Cb-FDH (2)	*Fused SMO co-expressed with FDH*	pET28b/pET21a	N-His_6 (1)/No-Tag (2)	Ref. [2]	lyophilized whole cells	[2]
Sp(S)-EH	*Sphingomonas sp. HXN200*	pET28b	N-His_6	1 mM IPTG, 25 °C 16 h	lyophilized whole cells	[3]
St(R)-EH	*Solanum tuberosum*	pET28b	N-His_6	1 mM IPTG, 25 °C 16 h	lyophilized whole cells	[3]
Aa-ADH	*Aromatoleum aromaticum*	pET28b	N-His_6	Ref. [4]	purified	[5]
Ls-ADH	*Leifsonia sp.*	pET21a	no Tag	0.5 mM IPTG, 25 °C 16 h	purified	[6]
Bs-BDHA	*Bacillus subtilis BGSC1A1*	pET28b	N-His_6	0.5 mM IPTG, 25 °C 16 h	purified	[7]
Bs-AlaDH	*Bacillus sphaericus*	pET28b-v	N-$Strep_3$	0.5 mM IPTG, 25 °C 16 h	purified	[8]
Cv(S)-ωTA	*Chromobacterium violaceum DSM 30191*	pET28b-v	N-His_6	0.5 mM IPTG, 25 °C 16 h	purified	[9]
At(R)-ωTA	*Aspergillus terreus*	pET21a	C-His_6	0.5 mM IPTG, 25 °C 16 h	purified	[10], [11]
Ac(S)-ωTA	*Arthrobacter citreus*	pET28b-v	N-His_6	0.5 mM IPTG, 25 °C 16 h	purified	[12]
Bm(S)-ωTA	*Bacillus megaterium SC6394*	pET28b-v	N-His_6	0.5 mM IPTG, 25 °C 16 h	purified	[13], [12]
Vf(S)-ωTA	*Vibrio fluvialis*	pET28b-v	N-His_6	0.5 mM IPTG, 25 °C 16 h	lyophilized whole cells	[14], [15]
As(R)-ωTA	*Arthrobacter species*	pET21a	C-His_6	0.5 mM IPTG, 25 °C 16 h	purified	[16]

2.1 General procedures for expression and purification of enzymes

Note: in the case of transaminases, PLP (0.5 mM) was added to the cell lysate before loading on the column; protein concentration was determined according to Bradford assay using Ovalbumin as standard protein.[17]

Expression of the enzymes: For recombinant expression, 800 mL of LB medium supplemented with the appropriate antibiotic (100 µg mL^{-1} ampicillin or 50 µg mL^{-1} kanamycin) were inoculated with 15 mL of an overnight culture harbouring the desired vector with genes for the expression of the enzymes. *E. coli* BL21 DE3 cells were used as host organism. Cells were grown at 37 °C until an OD_{600} of 0.6 to 1 was reached and the expression of the protein was induced by the addition of IPTG. Protein expression was carried out overnight and after harvesting of the cells by centrifugation (4 °C, 4500 rpm, 10 min), the remaining cell pellets were washed with buffer (for lyophilized cells: 50 mM Tris-HCl buffer, pH 8.0 for ADHs; 50 mM KPi, pH 8.0 for EHs and TAs; or lysis buffer for enzymes that were subsequently purified by affinity chromatography).

Purification by Ni^{2+} affinity chromatography: His_6-tagged proteins were resuspended in lysis buffer (50 mM KH_2PO_4, 300 mM NaCl, 10 mM imidazole, pH 8.0) prior to cell disruption, and protein purification was performed by Ni-NTA affinity chromatography using pre-packed Ni-NTA HisTrap HP columns (GE Healthcare), previously equilibrated with lysis buffer. After loading of the filtered lysate, the column was washed with sufficient amounts of washing buffer (50 mM KH_2PO_4, 300 mM NaCl, 25 mM imidazole, pH 8.0), and bound protein was recovered with elution buffer (50 mM KH_2PO_4, 300 mM NaCl, 200 mM imidazole, pH 8.0). Purity was analysed by SDS-PAGE and fractions showing >95% purity were combined and dialyzed overnight against Tris-HCl buffer (6 L, pH 8.0,

20 mM) or KPi buffer (6 L, pH 8.0, 50 mM). The enzyme solutions were concentrated and their concentration was determined spectrophotometrically based on their extinction coefficient at 280 nm.

Purification by Strep affinity chromatography: Strep-tagged proteins were resuspended in binding buffer (100 mM Tris-HCl, 150 mM NaCl and 1 mM EDTA, pH 8.0) prior to cell disruption, and protein purification was performed by Strep affinity chromatography using pre-packed columns with StrepTactin Sepharose (GE Healthcare), previously equilibrated with binding buffer. After washing of the column, the tagged protein was recovered with elution buffer (100 mM Tris-HCl, 150 mM NaCl, 1 mM EDTA and 2.5 mM desthiobiotin, pH 8.0). Purity was analysed by SDS-PAGE and fractions showing >95% purity were combined and dialyzed overnight against Tris-HCl buffer (6 L, pH 8.0, 20 mM). The enzyme solutions were concentrated and their concentration was determined spectrophotometrically based on their extinction coefficient at 280 nm.

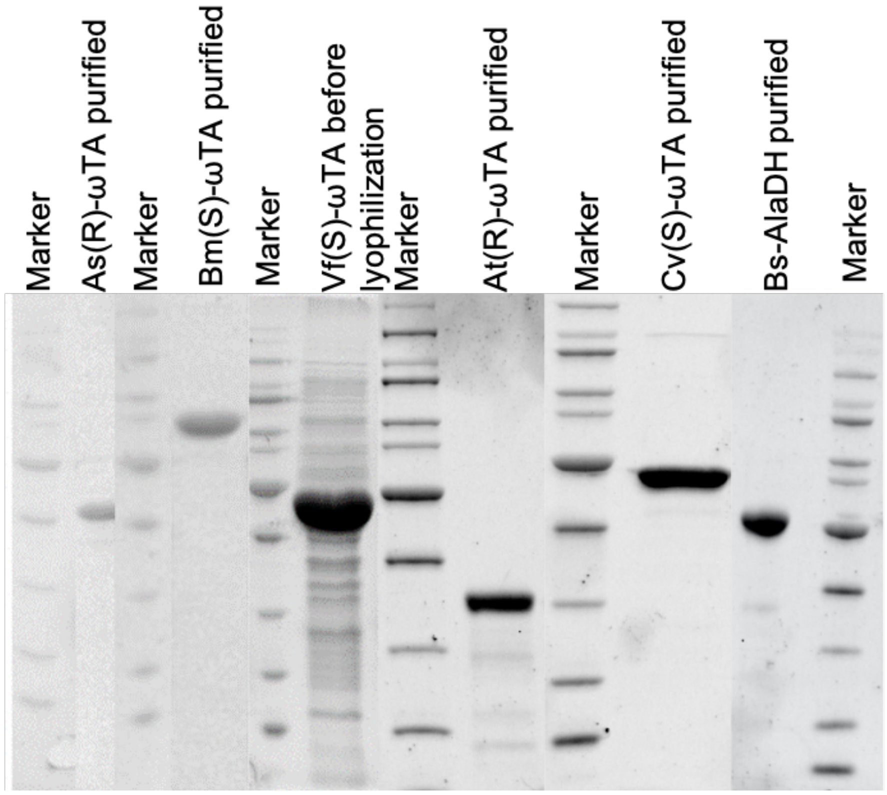

Figure S1. SDS-PAGE of lyophilized whole cells (a sample of crude cell extract prepared before lyophilization of the cells) or purified enzymes. Marker: PageRuler™ Unstained Protein Ladder (ThermoFisher Scientific).

3. Optimization of the one-pot cascade for the conversion of chiral diols 3 to optically active amino alcohols 5 and 5' by ADHs coupled with ω-transaminases

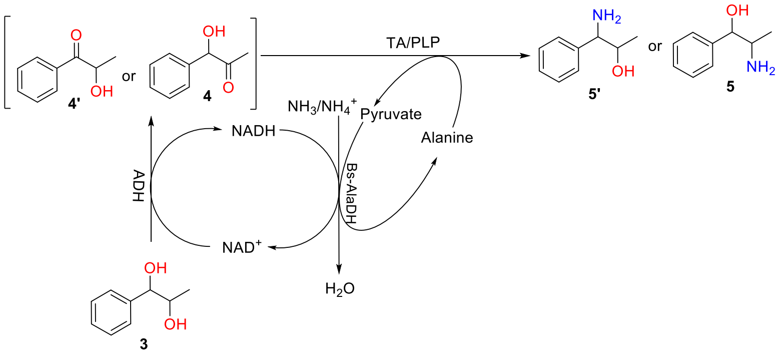

3.1 *General procedure for the one-pot cascade for the conversion of enantiopure diols **3** catalyzed by ADHs combined with ωTAs*

$HCOONH_4$ buffer (0.5 mL, pH 8.5, 1 M) supplemented with NAD^+ (1 mM), PLP (1mM) and D or L-alanine (5 eq.) was added to an Eppendorf tube (1.5 mL) followed by *Bs*-AlaDH (20 µM), ADH (35-70 µM, specified in each table caption) and ωTA (35-70 µM, specified in each table caption). Chiral substrate **3** (10-20 mM, specified in each table caption) was added as last. The reaction mixture was incubated at 30 °C (otherwise stated), 170 rpm for 48 h on an orbital shaker. The mixture was then quenched with 10 M KOH (100 µL), the aqueous layer saturated with solid NaCl and the extraction was performed with MTBE (1 x 500 µL). The organic layer was dried with $MgSO_4$ and analyzed by GC-FID (method A). Before quenching the reaction mixture, an aliquot of the aqueous phase was derivatized with GITC (i.e., 2,3,4,6-Tetra-O-acetyl-β-D-glucopyranosyl isothiocyanate) and analyzed by RP-HPLC (method B).

General procedure for the derivatization of the amino alcohol products to determine *er* and *dr* [18]. The aqueous reaction mixture (20 µL) was dissolved in acetonitrile (180 µL) to yield a final concentration of 0.5 mM. Then, GITC (1.5 mM) and Et_3N (1.5 mM) were added as a solution in acetonitrile (200 µL). The mixture was incubated at room temperature at 1000 rpm for 35 min. Before injection into the RP-HPLC, the samples were centrifuged and filtered if required.

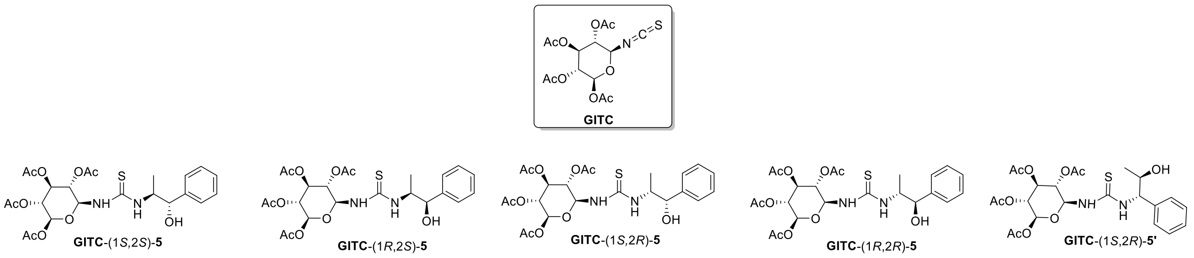

3.2 *Temperature study for the conversion of (1S,2S)-**3** catalyzed by Aa-ADH combined with Cv(S)-ωTA or At(R)-ωTA*

Substrate (1*S*,2*S*)-**3** (5 mM) was chosen as model substrate and Aa-ADH was then combined with Cv(*S*)-ωTA (20 °C, 30 °C, 40 °C and 50 °C) or At(*R*)-ωTA (30 °C, 40 °C and 50 °C). Both, ADH and ωTA were used in equimolar ratio (50:50 µM). The reactions were carried out in $HCOONH_4$ buffer (pH 8.5, 1 M, 0.5 mL) supplemented with NAD^+ (1 mM), PLP (1 mM), Bs-AlaDH (20 µM), D or L-alanine (25 mM, 5 eq.). The best temperature to perform this cascade reaction seems to be 30 °C, which will be used in all further experiments.

Table S2. Conversion [%] of (1*S*,2*S*)-**3** (5 mM) in the one-pot cascade catalyzed by Aa-ADH (50 µM) combined with two stereocomplementary ωTAs (50 µM) in $HCOONH_4$ buffer (pH 8.5, 1 M) at various temperatures.

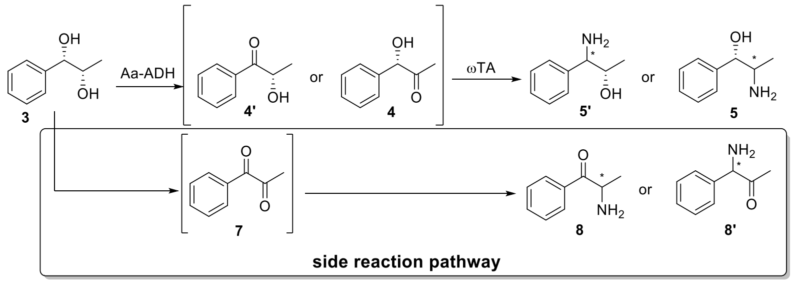

Entry	ωTA	T [°C]	Conv. [%]	5 [%]	*dr* [%][a]	4 [%]	4' [%]	5' [%]	7 [%]	8 [%]	8' [%]
1	Cv(*S*)	20	97±1	**67±1**	n.d.	7±<1	5±<1	n.d.	7±<1	3±<1	n.d.
2	Cv(*S*)	30	>99	**54±8**	99:1	13±5	12±3	n.d.	7±<1	10±1	n.d.
3	At(*R*)	30	>99	**92±1**	99:1	1±<1	1±<1	n.d.	1±<1	3±<1	n.d.
4	Cv(*S*)	40	94±2	**58±3**	73:27	8±1	9±1	n.d.	4±1	13±1	n.d.
5	At(*R*)	40	>99	**88±<1**	99:1	2±<1	2±<1	n.d.	1±<1	4±<1	n.d.
6	Cv(*S*)	50	96±1	**29±1**	41:59	20±<1	19±<1	n.d.	9±1	16±<1	n.d.
7	At(*R*)	50	95±<1	**53±1**	99:1	13±<1	11±<1	n.d.	7±<1	8±<1	n.d.

[a] Analyzed by GC-FID; Reactions were performed in duplicate and the reported results are the average of two samples.

*3.3 Screening of Aa-ADH with a panel of ω-transaminases with substrate (1S,2S)-**3***

The first screening of a panel of different ωTAs was performed with substrate (1*S*,2*S*)-**3** (20 mM). Aa-ADH (70 µM) was combined with five purified stereocomplementary ωTAs (35 µM); only Vf(*S*)-ωTA was used as lyophilized *E.coli* whole cells (20 mg mL^{-1}). The biotransformations were performed under the general conditions reported in section 3.1.

Table S3. One pot bio-catalytic conversion of (1*S*,2*S*)-**3** (20 mM) catalyzed by Aa-ADH (70 µM) combined with stereocomplementary ωTAs (35 µM) in $HCOONH_4$ buffer (pH 8.5, 1 M, 0.5 mL) and 30 °C.

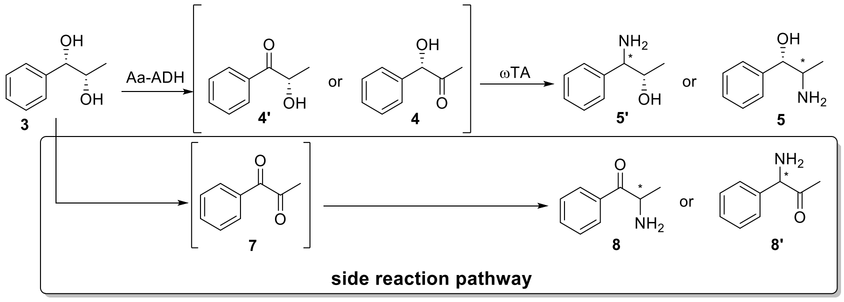

Entry	ωTA	Conv. [%]	4' [%]	4 [%]	5 [%]	*er* 5 [%][b]	*dr* 5 [%][b]	5' [%]	7 [%]	8 [%]	8' [%]
1	Cv(*S*)	94±1	2±<1	4±1	**86±<1**	86:14 [**SS**:RR]	93:7 [**SS**:RS]	n.d.	<1	2±<1	n.d.
2	Vf(*S*)[a]	48±10	9±1	15±2	**11±4**	n.m.	n.m.	n.d.	<1	13±2	n.d.
3	Bm(*S*)	91±<1	2±<1	3±<1	**86±<1**	86:14 [**SS**:RR]	95:5 [**SS**:RS]	n.d.	<1	1±<1	n.d.
4	Ac(*S*)	14±<1	1±<1	6±<1	**7±<1**	n.m.	n.m.	n.d.	<1	n.d.	n.d.
5	At(*R*)	>99	<1	1±<1	**96±<1**	99:1 [**SR**:RS]	2:98 [RR:**SR**]	n.d.	<1	1±<1	n.d.
6	As(*R*)	98±<1	1±<1	2±<1	**90±1**	99:1 [**SR**:RS]	4:96 [RR:**SR**]	n.d.	<1	2±<1	n.d.

[a] Used as lyophilized *E.coli* whole cells (20 mg mL^{-1}); [b] Determined by RP-HPLC (C18 column) after derivatization of the amino group with a chiral reagent (GITC); reactions were performed in duplicate and the reported results are the average of two samples.

*3.4 Screening of Ls-ADH and Aa-ADH with a panel of ω-transaminases with substrate (1R,2R)-**3***

Ls-ADH (35 µM) was combined with a panel of stereocomplementary ωTAs (70 and 50 µM, respectively) for the conversion of (1*R*,2*R*)-**3** (20 mM). The biotransformations were performed under the general conditions reported in section 3.1.

Table S4. One pot bio-catalytic conversion of (1*R*,2*R*)-**3** (20 mM) catalyzed by Ls-ADH (35 µM) combined with stereocomplementary ωTAs (70 µM) in $HCOONH_4$ (pH 8.5, 1 M, 0.5 mL) and 30 °C.

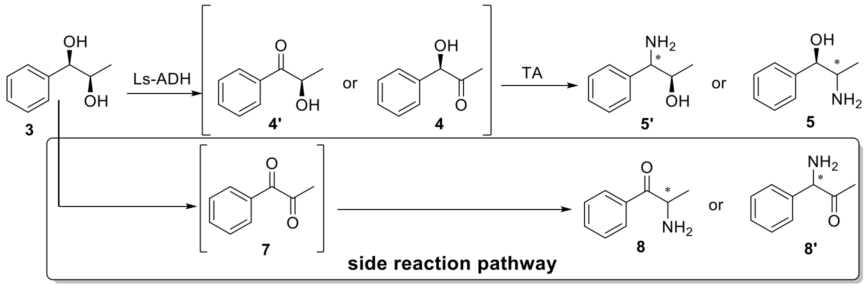

Entry	ωTA	Conv. [%]	4' [%]	4 [%]	5 [%]	*er* 5 [%][b]	*dr 5* [%][b]	5' [%]	7 [%]	8 [%]	8' [%]
1	Cv(*S*)	88±<1	1±<1	4±<1	**76±<1**	>99.5:<0.5 [**RS**:SR]	>99.5:<0.5 [**RS**:RR/SS]	n.d.	<1	7±<1	n.d.
2	Vf(*S*)[a]	>99	13±2	54±1	**n.d.**	n.m.	n.m	n.d.	n.d.	33±3	n.d.
3	Bm(*S*)	90±<1	1±<1	3±<1	**81±<1**	>99.5:<0.5 [**RS**:SR]	96:2:2 [**RS**:SS/RR]	n.d.	<1	5±<1	n.d.
4	Ac(*S*)	33±<1	3±<1	7±<1	**22±<1**	n.m.	n.m.	n.d.	2±<1	2±<1	n.d.
5	At*(R)*	>99	n.d.	2±<1	**95±<1**	>99.5:<0.5 [**RR**:SS]	>99.5:<0.5 [**RR**:SR/RS]	n.d.	n.d.	2±<1	n.d.
6	As*(R)*	>99	n.d.	4±<1	**90±<1**	>99.5:<0.5 [**RR**:SS]	>99.5:<0.5 [**RR**:SR/RS]	n.d.	<1	6±<1	n.d.

[a] Used as lyophilized whole *E.coli* cells (20 mg mL^{-1}); [b] Determined by achiral RP-HPLC (C18 column) after derivatization of the amino group with a chiral reagent (GITC); Reactions were performed in duplicate and the reported results are the average of two samples.

Based on the preliminary study in which the formation of the regioisomer **5'** was observed, (1*R*,2*R*)-**3** (10 mM) was tested as substrate for the enzymatic one-pot cascade performed by Aa-ADH (50 µM) combined with the stereocomplementary ωTAs (50 µM). The biotransformations were performed under the general conditions reported in section 3.1.

Table S5. One pot bio-catalytic conversion of (1*R*,2*R*)-**3** (10 mM) catalyzed by Aa-ADH (50 µM) combined with stereocomplementary ωTAs (50 µM) in $HCOONH_4$ (pH 8.5, 1 M, 0.5 mL) and 30 °C.

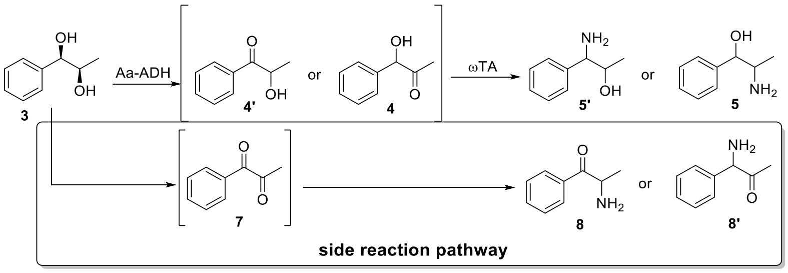

Entry	ωTA	Conv. [%]	4' [%]	4 [%]	5' [%]	*er* 5' [%][b]	*dr* 5' [%][b]	5 [%]	*er* 5 [%][b]	*dr* 5 [%][b]	7 [%]	8 [%]	8' [%]
1	Cv(*S*)	47±1	15±1	3±1	**n.d.**	n.m.	n.m.	**24±1**	>99.5:<0.5 [**RS**:SR]	>99.5:<0.5 [**RS**:SS/RR]	<1	5±<1	n.d.
2	Vf(*S*)[a]	76±7	61±6	10±1	**n.d.**	n.m.	n.m.	**24±7**	n.m.	n.m.	<1	3±<1	1±<1
3	Bm(*S*)	47±<1	14±1	2±<1	**n.d.**	n.m.	n.m.	**26±<1**	>99.5:<0.5 [**RS**:SR]	14:86 [RR:**RS**]	<1	5±<1	n.d.
4	Ac(*S*)	20±1	9±1	3±<1	**n.d.**	n.m.	n.m.	**7±<11**	n.m.	n.m.	2±<1	1±<1	n.d.
5	At(*R*)	97±1	2±<1	n.d.	**58±1**	>99.5:<0.5 [**SR**:RS]	95:5 [**SR**:RR]	**21±<1**	>99.5:<0.5 [**RR**:SS]	>99.5:<0.5 [**RR**:SR/RS]	n.d.	1±<1	13±<1
6	As(*R*)	94±1	3±2	n.d.	**61±<1**	>99.5:<0.5 [**SR**:RS]	>99.5:<0.5 [**SR**:RR/SS]	**16±<1**	>99.5:<0.5 [**RR**:SS]	>99.5:<0.5 [**RR**:SR/RS]	<1	2±<1	10±<1

[a] Used as lyophilized *E.coli* whole cells (20 mg mL^{-1}); [b] determined by RP-HPLC (C18 column) after derivatization of the amino group with a chiral reagent (GITC); reactions were performed in duplicate and the reported results are the average of two samples.

*3.5 Screening of Bs-BDHA with a panel of ω-transaminases with substrate (1S,2R)-**3***

The same screening was then carried out on substrate (1*S*,2*R*)-**3** (15 mM) by combining in one-pot Bs-BDHA (50 µM) with a panel of stereocomplementary ωTAs (50 µM). The biotransformations were performed under the general conditions reported in section 3.1.

Table S6. One pot bio-catalytic conversion of (1*S*,2*R*)-**3** (15 mM) catalyzed by Bs-BDHA (50 µM) coupled with stereocomplementary ωTAs (50 µM) in $HCOONH_4$ (pH 8.5, 1 M) and 30 °C.

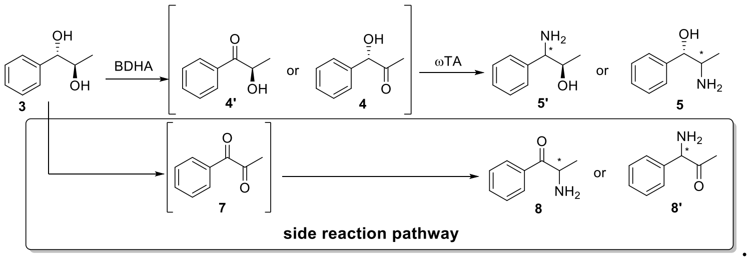

Entry	ωTA	Conv. [%]	4' [%]	4 [%]	5' [%]	5 [%]	*er* 5 [%][b]	*dr* [%][b]	7 [%]	8 [%]	8' [%]
1	Cv(*S*)	92±3	1±<1	3±<1	n.d.	**86±3**	>99.5:<0.5 [**SS**:RR]	96:4 [**SS**:RS]	<1	3±1	n.d.
2	Vf(*S*)[a]	29±<1	6±<1	11±<1	n.d.	**3±<1**	n.m.	n.m.	<1	8±1	n.d.
3	Bm(*S*)	94±1	1±<1	2±<1	n.d.	**88±1**	>99.5:<0.5 [**SS**:RR]	96:4 [**SS**:RS]	<1	3±<1	n.d.
4	Ac(*S*)	16±<1	<1	4±<1	n.d.	**6±<1**	n.m.	n.m.	n.d.	<1	n.d.
5	At(*R*)	98±2	1±<1	1±<1	n.d.	**95±2**	>99.5:<0.5 [**SR**:RS]	98:2 [**SR**:RR]	<1	2±<1	n.d.
6	As(*R*)	97±1	1±<1	1±<1	n.d.	**92±<1**	>99.5:<0.5 [**SR**:RS]	97:3 [**SR**:RR]	<1	3±<1	n.d.

[a] Used as lyophilized *E.coli* whole cells (20 mg mL^{-1}); [b] determined by RP-HPLC (C18 column) after derivatization of the amino group with a chiral reagent (GITC); reactions were performed in duplicate and the reported results are the average of two samples.

*3.6 Screening of Aa-ADH with a panel of ω-transaminases with substrate (1R,2S)-**3***

As last, the screening was performed with substrate (1*R*,2*S*)-**3** (10 mM) by combining in one-pot Aa-ADH (70 µM) with a panel of stereocomplementary ωTAs (35 µM). The biotransformations were performed under the general conditions reported in section 3.1.

Table S7. One pot bio-catalytic conversion of (1*R*,2*S*)-**3** (10 mM) catalyzed by Aa-ADH (70 µM) combined with stereocomplementary ωTAs (35 µM) in $HCOONH_4$ (pH 8.5, 1 M, 0.5 mL) and 30 °C.

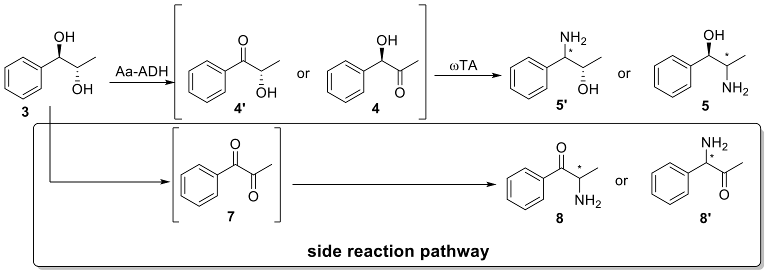

Entry	ωTA	Conv. [%]	4' [%]	4 [%]	5' [%]	5 [%]	*er* 5 [%][b]	*dr* 5 [%][b]	7 [%]	8 [%]	8' [%]
1	Cv(*S*)	92±1	3±<1	1±<1	n.d.	**77±<1**	>99.5:<0.5 [**RS**:SR]	17:83 [RR:**RS**]	2±<1	10±1	n.d.
2	Vf(*S*)[a]	99±1	13±6	33±10	1±1	**4±4**	n.m.	n.m.	1±<1	45±11	n.d.
3	Bm(*S*)	87±1	2±<1	1±<1	n.d.	**76±<1**	>99.5:<0.5 [**RS**:SR]	31:69 [RR:**RS**]	3±1	7±1	n.d.
4	Ac(*S*)	23±1	5±<1	5±<1	n.d.	**10±<1**	n.m.	n.m.	2±<1	1±<1	n.d.
5	At(*R*)	>99	n.d.	n.d.	5±<1	**87±<1**	>99.5:<0.5 [**RR**:SS]	70:30 [**RR**:RS]	n.d.	4±<1	4±<1
6	As(*R*)	>99	1±<1	<1	1±<1	**84±<1**	>99.5:<0.5 [**RR**:SS]	83:17 [**RR**:RS]	1±<1	13±<1	n.d.

[a] Used as lyophilized *E.coli* whole cells (20 mg mL^{-1}); [b] determined by RP-HPLC (C18 column) after derivatization of the amino group with a chiral reagent (GITC); reactions were performed in duplicate and the reported results are the average of two sample

4. Preparative scale reactions

4.1 One-pot biocatalytic synthesis of enantiomerically pure diols (1S,2R)-3 and (1R,2R)-3 from cis- and trans-β-methylstyrene at hundreds of milligrams scale

General remark: The concentrations of coenzymes and co-substrate are always calculated on the volume of the aqueous phase, whereas the concentration of the substrate is referred to the organic phase.

Reaction conditions: Lyophilized *E. coli* cells co-expressing Fus-SMO/Cb-FDH (250 mg, 5 mg mL^{-1}) were rehydrated in KPi buffer (50 mL, 50 mM, pH 8.0) in a baffled Erlenmeyer flask (500 mL). After that, NAD^+ (1 mM), HCOONa (5 eq.), FAD (50 µM) and catalase (0.1 mg mL^{-1}) were added. Heptane (50 mL; 1:1 volumetric ratio with the buffer) was used as biphasic solvent. Finally, the biocatalytic reactions were initiated by the addition of substrate *trans* or *cis*-**1** (50 mM, 2.5 mmol). The reactions were incubated at 30 °C and 200 rpm on an orbital shaker. After 5 hours, lyophilized *E. coli* cells expressing either Sp(S)-EH or St(R)-EH (1 g, 20 mg mL^{-1}) were added and the reactions were further incubated at 30 °C and 170 rpm on an orbital shaker for 30 h. Heptane was removed, the aqueous phase was saturated with solid NaCl and the organic compounds extracted with MTBE (3 x 50 mL). After drying over $MgSO_4$, the organic phase was removed under reduced pressure. The conversions and purity of the isolated products were determined by GC-FID, while *er* [%] and *dr* [%] were analysed by HPLC (Table S8).

Table S8: Preparative scale biocatalytic cascade for the conversion of *trans* or *cis*-**1** to enantiomerically pure **3** by combining Fus-SMO/Cb-FDH and two stereo-complementary EHs.

Entry	Substrate	EH	Isolated yield [%]	Purity [%][a]	*er* [%][b]	*dr* [%][b]
1	*trans*-**1**	Sp(*S*)-EH	86 (327 mg)	>98	99:1 (1*S*,2*R*-**3**)	>99.5:<0.5
2	*cis*-**1**	St(*R*)-EH	70 (266 mg)	>96	>99.5:<0.5 (1*R*,2*R*-**3**)	>99.5:<0.5

[a]determined by GC-FID; [b] analysed by HPLC

4.2 One-pot biocatalytic amination of (1R,2R)-3 and (1S,2R)-3 at hundreds of milligrams scale

The one-pot biocatalytic amination on preparative scale was performed for two out of the four enantiomerically pure or enantiomerically enriched diols **3** as reported in Table S8 by applying the previously identified optimal conditions. Conversions and isolated products were analyzed by GC-FID (method A), whereas enantiomeric and as diastereomeric ratios were determined by RP-HPLC (method B) after derivatization with GITC.

Reaction conditions for the biocatalytic amination of (1*R*,2*R*)-3 to yield (1*R*,2*S*)-5: To a 100 mL Erlenmeyer flask, $HCOONH_4$ buffer (65 mL final volume, pH 8.5, 1 M), supplemented with NAD^+ (1 mM), PLP (1 mM) and L-alanine (100 mM) was added followed by Bs-AlaDH (20 µM), Ls-ADH (35 µM) and Cv-ωTA (70 µM). Substrate (1*R*,2*R*)-**3** (20 mM, 202 mg, 1.3 mmol) was added as last. The mixture was incubated at 30 °C and 170 rpm on an orbital shaker for 48 h. The reaction was quenched with KOH (3 mL, 10 M). The aqueous layers were saturated with solid NaCl and the extraction was performed with MTBE (3 x 50 mL). The organic layers were then dried over $MgSO_4$ and concentrated under reduced pressure. Yield and selectivity data are reported in Table S8.

Reaction conditions for the biocatalytic amination of (1*S*,2*R*)-3 to yield (1*S*,2*S*)-5: To a 100 mL Erlenmeyer flask, $HCOONH_4$ buffer (44 mL final volume, pH 8.5, 1 M), supplemented with NAD^+ (1 mM), PLP (1 mM) and L-alanine (75 mM) was added followed by Bs-AlaDH (20 µM), Bs-BDHA (50 µM) and Bm-ωTA (50 µM). Substrate (1*S*,2*R*)-**3** (15 mM, 106 mg, 0.69 mmol) was added as last. The mixture was incubated at 30 °C and 170 rpm on an orbital shaker for 48 h. The reaction was quenched with KOH (3 mL, 10 M). The aqueous layers were saturated with solid NaCl and the extraction was performed with MTBE (3 x 50 mL). The organic layers were then dried over $MgSO_4$ and concentrated under reduced pressure. Yield and selectivity data are reported in Table S9.

Table S9. GC-FID and RP-HPLC analysis after isolation

**Entry**	**Sub.**	**ADH**	**ωTA**	**Conv. [%]**	**5 [%]**	***er* 5 [%][b]**	***dr 5* [%][b]**	**8 [%]**
1	1*R*,2*R*-**3**	Ls-ADH	Cv-ωTA	83	**75**	>99.5:<0.5 [**RS**:SR]	>99.5:<0.5 [**RS:**SS/RR]	8
2	1*S*,2*R*-**3**	Bs-BDHA	Bm-ωTA	86	**83**	>99.5:<0.5 [**SS**:RR]	97:3 [**SS**:RS]	3

5. Analytics

5.1 Methods

GC-FID Method A: Column: Agilent J&W DB1701 (30 m, 250 µm, 0.25 µm). Carrier gas: H_2 Parameter: T injector 250 °C; constant pressure 6.9 psi; temperature program: 80 °C, hold 6.5 min; gradient 5 °C min^{-1} up to 160 °C, hold 5 min; gradient 20 °C min^{-1} up to 200 °C, hold 2 min; gradient 20 °C min^{-1} up to 280 °C, hold 4 min.

RP-HPLC Method B: Column: Nucleosil C_{18} HD (0.46 cm x 25 cm): HPLC program: constant oven temperature 30 °C; eluent composition: isocratic 50:50 (v v^{-1}) MeOH:MilliQ (+ 0.1% TFA); flow rate: 1 mL min^{-1}, detection at 248 nm.

Table S10. Retention times and associated analytical methods

Compound	Retention time [min]	Method
(1*S*,2*R*)-**5'**	22.3	A
	13.8	B[a]
(1*R*,2*S*)-**5'**	22.3	A
	17.2	B
(1*R*,2*R*)-**5'**	21.9	A
	15.7	B
(1*S*,2*S*)-**5'**	21.9	A
	18.5	B
**8'**	21.0	A

[a]After derivatization with GITC. Retention times of chiral substrates **3** (all four isomers), chiral amino alcohols **5** (all four isomers), compounds **4**, **4'**, **7** and **8** and associated methods were reported previously.[1]

5.2 *Representative GC-FID and RP-HPLC spectra*

- Conversion of (1*R*,2*R*)-**3** catalyzed by Aa-ADH combined with As(*R*)-ωTA

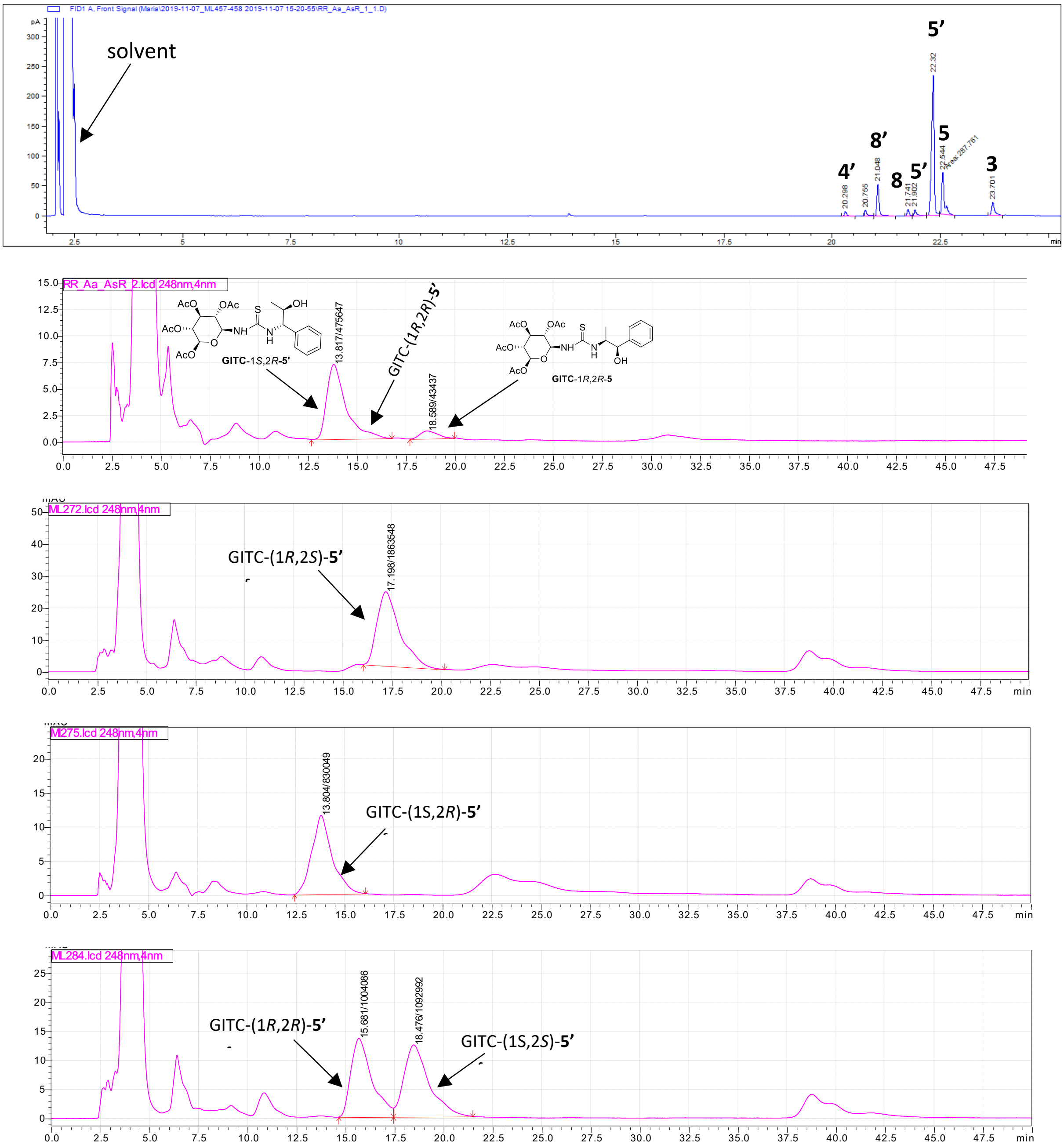

- Conversion of (1*R*,2*R*)-**3** catalyzed by Ls-ADH combined with At(*R*)-ωTA

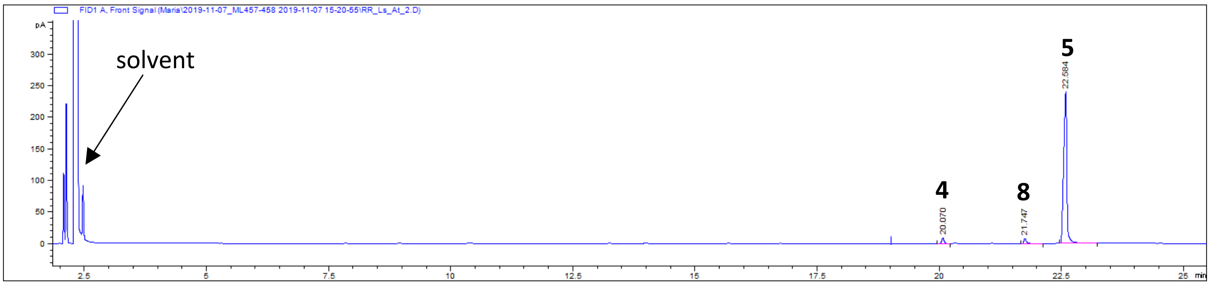

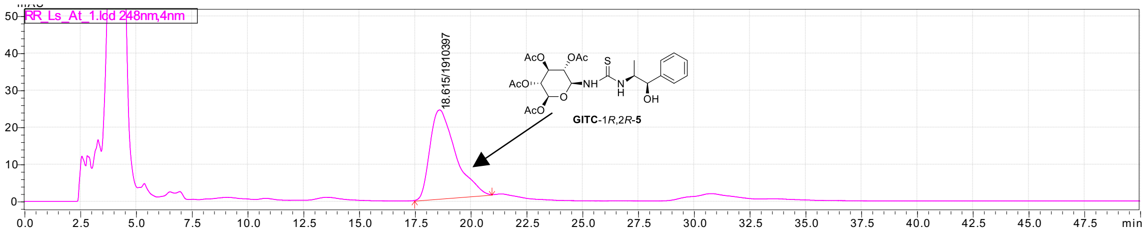

- Conversion of (1*R*,2*R*)-**3** catalyzed by Ls-ADH combined with Bm(*S*)-ωTA

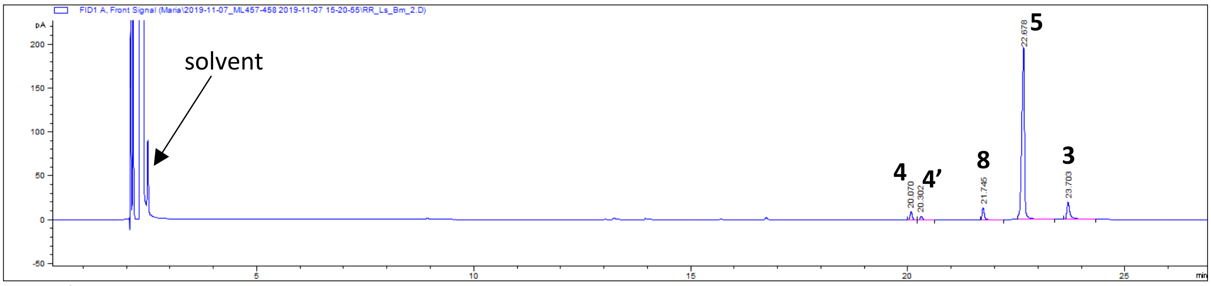

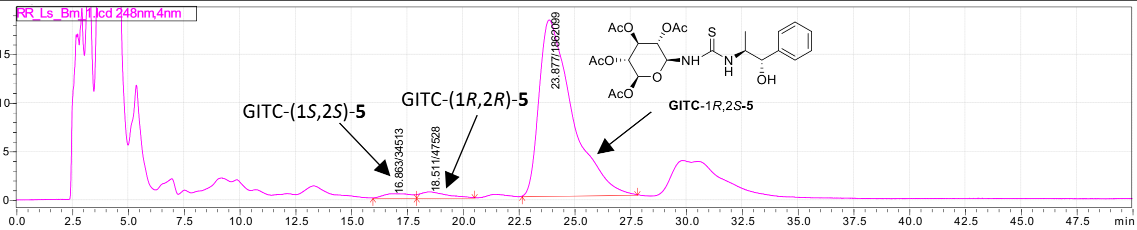

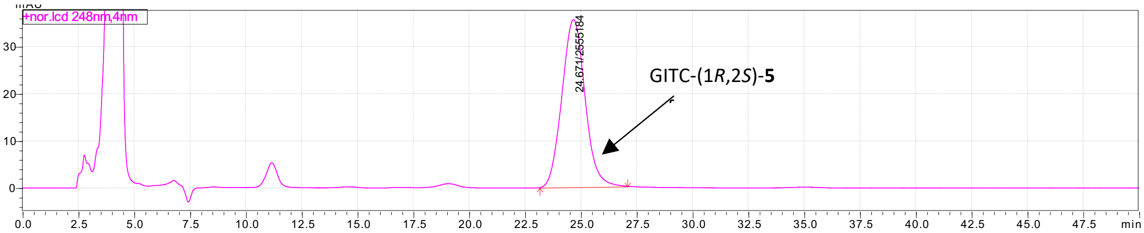

- Conversion of (1*S*,2*R*)-**3** catalyzed by Bs-BDHA combined with At(*R*)-ωTA

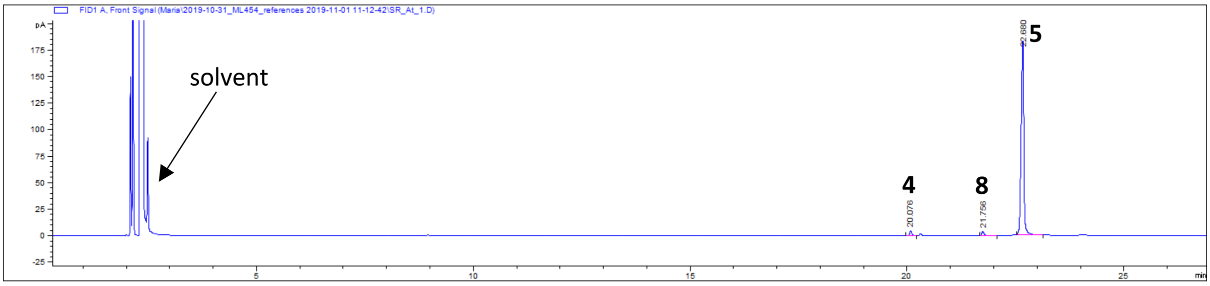

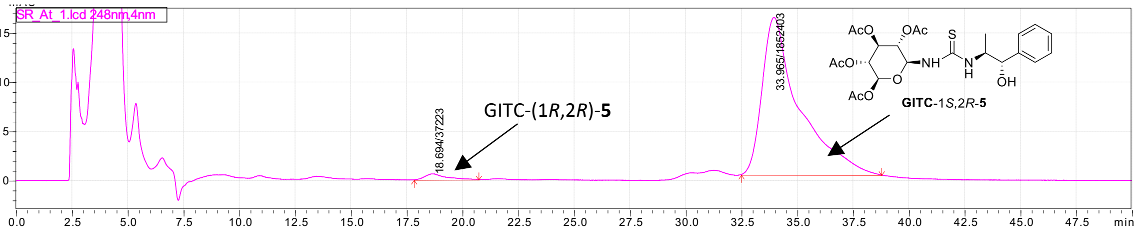

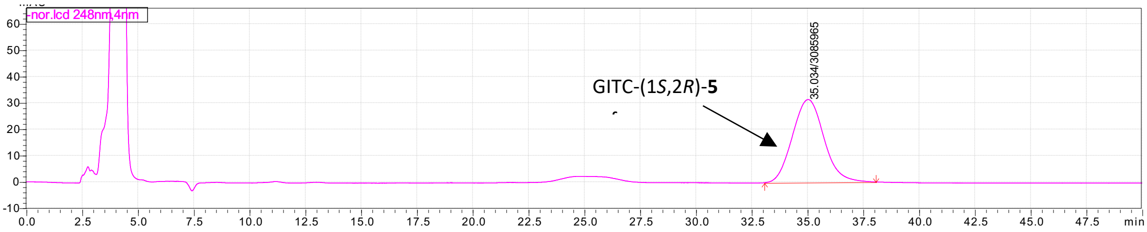

- Conversion of (1*S*,2*R*)-**3** catalyzed by Bs-BDHA combined with Cv(*S*)-ωTA

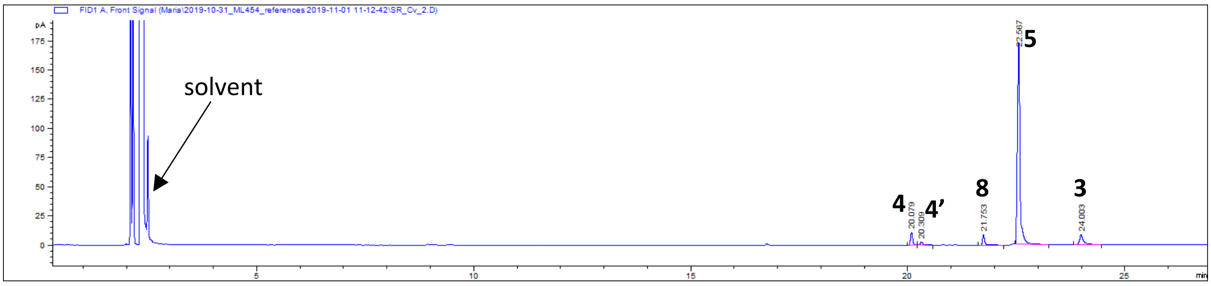

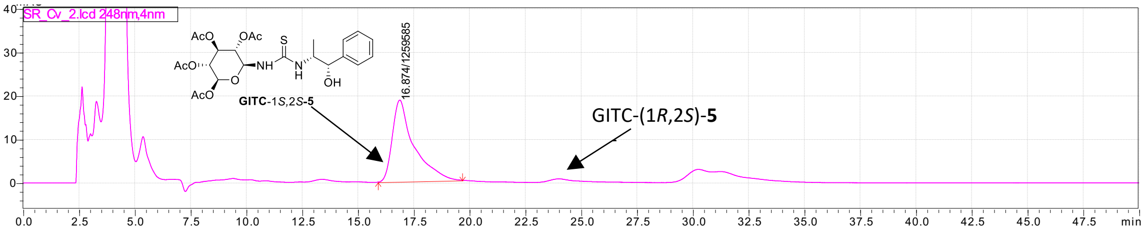

6. References

[1] M. L. Corrado, T. Knaus, F. G. Mutti, *Green Chem.* **2019**, *21*, 6246-6251.

[2] M. L. Corrado, T. Knaus, F. G. Mutti, *ChemBioChem* **2018**, *19*, 679-686.

[3] S. Wu, Y. Chen, Y. Xu, A. Li, Q. Xu, A. Glieder, Z. Li, *ACS Catal.* **2014**, *4*, 409-420.

[4] W. Bohmer, T. Knaus, F. G. Mutti, *ChemCatChem* **2018**, *10*, 731-735.

[5] H. W. Hoffken, M. Duong, T. Friedrich, M. Breuer, B. Hauer, R. Reinhardt, R. Rabus, J. Heider, *Biochemistry* **2006**, *45*, 82-93.

[6] K. Inoue, Y. Makino, N. Itoh, *Appl. Environ. Microbiol.* **2005**, *71*, 3633-3641.

[7] J. Zhang, T. Xu, Z. Li, *Adv. Synth. Catal.* **2013**, *355*, 3147-3153.

[8] T. Ohashima, K. Soda, *Eur. J. Biochem.* **1979**, *100*, 29-30.

[9] U. Kaulmann, K. Smithies, M. E. B. Smith, H. C. Hailes, J. M. Ward, *Enzyme Microb. Technol.* **2007**, *41*, 628-637.

[10] A. Lyskowski, C. Gruber, G. Steinkellner, M. Schurmann, H. Schwab, K. Gruber, K. Steiner, *PloS One* **2014**, *9*, e87350.

[11] F. G. Mutti, C. S. Fuchs, D. Pressnitz, J. H. Sattler, W. Kroutil, *Adv. Synth. Catal.* **2011**, *353*, 3227-3233.

[12] N. van Oosterwijk, S. Willies, J. Hekelaar, A. C. Terwisscha van Scheltinga, N. J. Turner, B. W. Dijkstra, *Biochemistry* **2016**, *55*, 4422-4431.

[13] R. L. Hanson, B. L. Davis, Y. Chen, S. L. Goldberg, W. L. Parker, T. P. Tully, M. A. Montana, R. N. Patel, *Adv. Synth. Catal.* **2008**, *350*, 1367-1375.

[14] F. G. Mutti, C. S. Fuchs, D. Pressnitz, N. G. Turrini, J. H. Sattler, A. Lerchner, A. Skerra, W. Kroutil, *Eur. J. Org. Chem.* **2012**, *2012*, 1003-1007.

[15] J. S. Shin, H. Yun, J. W. Jang, I. Park, B. G. Kim, *Appl. Microbiol. Biotechnol.* **2003**, *61*, 463-471.

[16] A. Iwasaki, Y. Yamada, N. Kizaki, Y. Ikenaka, J. Hasegawa, *Appl. Microbiol. Biotechnol.* **2006**, *69*, 499-505.

[17] W. Bohmer, T. Knaus, A. Volkov, T. K. Slot, N. R. Shiju, K. Engelmark Cassimjee, F. G. Mutti, *J. Biotechnol.* **2019**, *291*, 52-60.

[18] M. S. Malik, E.-S. Park, J.-S. Shin, *Green Chem.* **2012**, *14*, 2137.
